# Glucosinolate Distribution in the Aerial Parts of *sel1-10*, a Disruption Mutant of the Sulfate Transporter SULTR1;2, in Mature *Arabidopsis thaliana* Plants

**DOI:** 10.3390/plants8040095

**Published:** 2019-04-10

**Authors:** Tomomi Morikawa-Ichinose, Sun-Ju Kim, Alaa Allahham, Ryota Kawaguchi, Akiko Maruyama-Nakashita

**Affiliations:** 1 Department of Bioscience and Biotechnology, Faculty of Agriculture, Kyushu University, 744 Motooka, Nishi-ku, Fukuoka 819-0395, Japan; ichinose226@agr.kyushu-u.ac.jp (T.M.-I.); 2BE17488K@s.kyushu-u.ac.jp (A.A.); 2BE19462N@s.kyushu-u.ac.jp (R.K.); 2 Department of Bio-Environmental Chemistry, College of Agriculture and Life Sciences, Chungnam National University, Daejeon 34134, Korea; kimsunju@cnu.ac.kr

**Keywords:** mature *Arabidopsis thaliana* plants, sulfate transporter, SULTR1;2, *sel1-10* mutant, glucosinolates

## Abstract

Plants take up sulfur (S), an essential element for all organisms, as sulfate, which is mainly attributed to the function of SULTR1;2 in *Arabidopsis*. A disruption mutant of *SULTR1;2, sel1-10,* has been characterized with phenotypes similar to plants grown under sulfur deficiency (−S). Although the effects of −S on S metabolism were well investigated in seedlings, no studies have been performed on mature *Arabidopsis* plants. To study further the effects of −S on S metabolism, we analyzed the accumulation and distribution of S-containing compounds in different parts of mature *sel1-10* and of the wild-type (WT) plants grown under long-day conditions. While the levels of sulfate, cysteine, and glutathione were almost similar between *sel1-10* and WT, levels of glucosinolates (GSLs) differed between them depending on the parts of the plant. GSLs levels in the leaves and stems were generally lower in *sel1-10* than those in WT. However, *sel1-10* seeds maintained similar levels of aliphatic GSLs to those in WT plants. GSL accumulation in reproductive tissues is likely to be prioritized even when sulfate supply is limited in *sel1-10* for its role in S storage and plant defense.

## 1. Introduction

Sulfur (S) is an essential macronutrient for all organisms. Plants take up inorganic sulfate as the major S source and assimilate it into a variety of S-containing organic compounds [1,2]. As animals are unable to assimilate sulfate, the role of plants in the global S cycle on the earth is extremely important [2]. In addition, many of the S-containing compounds biosynthesized in plants are beneficial to health, such as methionine (an essential amino acid for animals), glutathione (a redox controller), and various secondary compounds with specific functions [2]. Glucosinolates (GSLs) are the major S-containing secondary compounds biosynthesized in *Brassicaceae*, that act as defense compounds against insects and pathogens [3,4,5]. Depending on their amino acid precursors, most GSLs accumulated in *Arabidopsis* are classified into aliphatic and indolic GSLs (iGSLs) synthesized from methionine and tryptophan, respectively [3,4,5]. Among them, some aliphatic GSLs (mGSLs) are known to be beneficial for humans as cancer-preventive chemicals [6,7]. Thus, understanding GSL accumulation in plant tissues would contribute to improved food quality in Brassica crops. 

The composition and content of GSLs are different among plant parts in *Arabidopsis* [8,9,10,11,12]. Most GSLs accumulated in developing rosette leaves are mGSLs, and mainly consist of 4-methylsulfinylbutyl GSL (4MSOB, 34 to 60%), 3-methylsulfinylpropyl GSL (3MSOP, 4 to 9%), 4-methylthiobutyl GSL (4MTB, 1 to 23%), and 8-methylsulfinyloctyl GSL (8MSOO, 2 to 6%). The remaining GSLs are iGSLs, and mostly comprise indol-3-ylmethyl GSL (I3M, 11 to 23%) [8,9,13]. Cauline leaves and stems have a similar concentration and composition to that of rosette leaves [9]. GSL content in the seeds is 3.5- to 8.5-fold than that in the leaves, with the higher GSL variations characterized by a higher amount of 4MTB (37 to 41%); the long-chain mGSLs, such as 8MSOO (9.9 to 10%), 8-methylthiooctyl GSL (8MTO, 6.9 to 7.4%), 7-methylthioheptyl GSL (7MTH, 4.7 to 4.8%), and 7-methylsulfinylheptyl GSL (7MSOH, 1.8 to 2.4%); as well as with a relatively low amount of I3M (2.3 to 2.9%) [8,9]. mGSLs are structurally divided into methylsulfinylalkyl (MSOX) GSLs (3MSOP, 4MSOB, 7MSOH, and 8MSOO) and methylthioalkyl (MTX) GSLs (4MTB, 7MTH, and 8MTO) [3,4,5,7]. Seeds accumulated more MTX GSLs than MSOX GSLs compared to the other tissues [8,9]. The GSL concentration in the siliques is lower than that in the seeds, and the composition is intermediate of that in the rosette leaves and the seeds [8,9]. This plant part-specific variation in GSL concentration and composition suggests that GSL accumulation is controlled by different mechanisms in each part [8,9,10,11,12].

GSL content in plants is also influenced by environmental factors [5,14]. For example, it is stimulated by glucose and jasmonic acid [15,16], and is increased upon pathogen infection [17,18] and insect bite [19]. Among the environmental factors, nutritional conditions, particularly S status, greatly influence GSL accumulation in plants [13,20,21,22]. GSL synthesis and accumulation are stimulated under S sufficiency (+S) but suppressed under S deficiency (−S), which is regulated by specific transcriptional networks induced by −S in *Arabidopsis* [5,13,20,22,23,24]. However, these experiments were mostly undertaken on seedlings and the effects of –S on GSL accumulation in mature plants have not been reported. 

Previous studies have shown a close correlation between the effects of −S and the disruption of SULTR1;2, a major sulfate transporter that facilitates sulfate uptake from roots [25,26,27,28]. In this study, we examined the accumulation of S-containing compounds in aerial tissues of mature SULTR1;2 mutants, known as *sel1-10*, and wild-type (WT) plants to clarify the distribution of sulfate as well as cysteine and glutathione in relation to the distribution of GSL in the mature plants. 

## 2. Results

### 2.1. Growth Phenotypes of WT and sel1-10 Plants 

**Figure 1:**
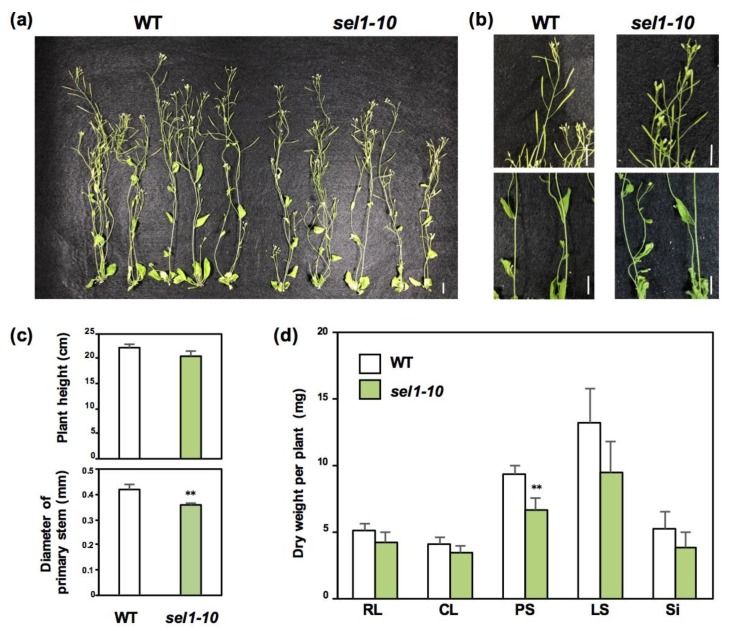
Growth phenotypes of wild-type (WT) and *sel1-10* plants. (**a**) WT and *sel1-10* plants grown for six weeks on vermiculite. (**b**) Siliques (upper panels) and primary stems (lower panels) of WT and *sel1-10* plants. Scale bar = 1 cm. (**c**) Plant heights and diameters of primary stems of WT and *sel1-10* plants. (**d**) Dry weight of rosette leaves (RL), cauline leaves (CL), primary stems (PS), lateral stems (LS), and siliques (Si) in WT and *sel1-10* plants. White and green bars represent WT and *sel1-10*, respectively, in (**c**) and (**d**). Data are shown as the averages with error bars denoting SEM (n = 5). Asterisks indicate significant differences (Student’s *t*-test; ** *p* < 0.05) between WT and *sel1-10*.

To investigate the metabolic changes occurring in mature *sel1-10* plants, we initially observed the growth phenotypes of *sel1-10* plants (Figure 1). WT and *sel1-10* plants were grown for six weeks in vermiculite. Although visible differences in shoot phenotype were not observed between WT and *sel1-10* plants (Figure 1a,b), a significant decrease was observed in the primary stem diameters of *sel1-10* plants compared to those of the WT, while the plant heights were similar between WT and *sel1-10* plants (Figure 1c). Correlated with the decrease in primary stem diameter in *sel1-10*, dry weight of primary stems (PS) was decreased in *sel1-10* to 70% of that in WT plants (Figure 1d). Dry weights of rosette leaves (RL), cauline leaves (CL), lateral stems (LS), and siliques (Si) were not significantly lower but tended to be lower in *sel1-10* plants relative to those in WT plants (Figure 1d).

### 2.2. Concentrations of Sulfate and Selected Sulfur-Containing Metabolites in Different Parts of WT and sel1-10 Plants

**Figure 2:**
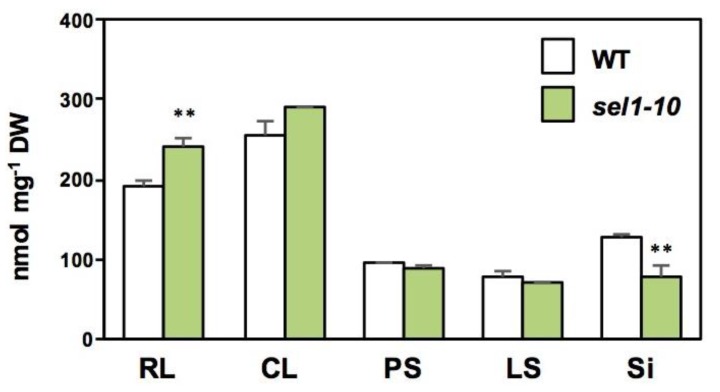
Sulfate concentrations in different parts of WT and *sel1-10* plants. Sulfate content in each part was determined by ion chromatography. WT and *sel1-10* seedlings were grown for six weeks in vermiculite, and each part was harvested. Rosette leaves (RL), cauline leaves (CL), primary stems (PS), lateral stems (LS), and siliques (Si). White and green bars represent the sulfate content in WT and *sel1-10*, respectively. Data are shown as averages with error bars denoting SEM (n = 3). Asterisks indicate significant differences (Student’s *t*-test; * 0.05 < *p* < 0.1, ** *p* < 0.05) between WT and *sel1-10* plants.

**Figure 3:**
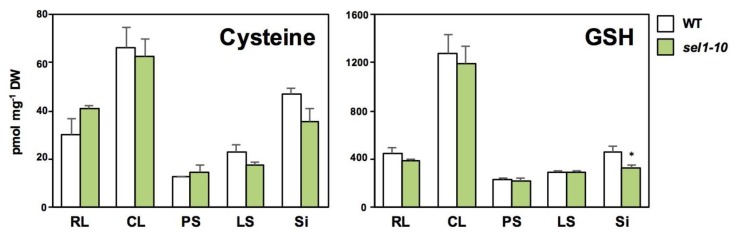
Cysteine and glutathione (GSH) concentrations in different parts of WT and *sel1-10* plants. The cysteine and GSH contents of different parts were measured using HPLC-fluorescence detection. WT and *sel1-10* seedlings were grown for 6 weeks in vermiculite, after which each part was harvested. Rosette leaves (RL), cauline leaves (CL), primary stems (PS), lateral stems (LS), and siliques (Si). White and green bars represent cysteine and GSH levels in WT and *sel1-10* plants, respectively. Data are shown as the averages with error bars denoting SEM (n = 3). Asterisks indicate significant differences (Student’s *t*-test; * 0.05 < *p* < 0.1, ** *p* < 0.05) between WT and *sel1-10* plants.

**Figure 4:**
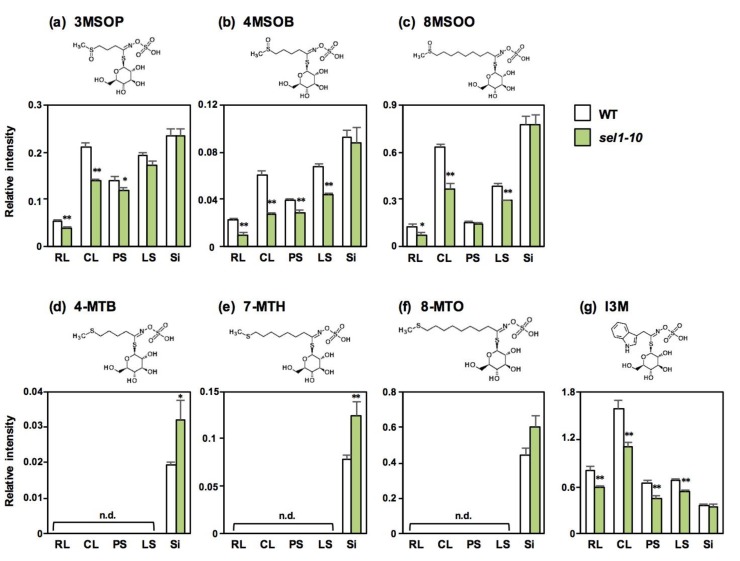
Glucosinolates (GSL) accumulation in different parts of WT and *sel1-10* plants. GSL levels in different parts were determined by LC-MS. The relative amount was calculated as the ratio of peak height of each GSL to that of the internal standard (L(+)-10-camphor sulfonic acid) and then divided by the dry weight of the sample. WT and *sel1-10* seedlings were grown for 6 weeks in vermiculite, after which, each part was harvested. (**a**) 3-methylsulfinylpropyl GSL (3MSOP), (**b**) 4-methylsulfinylbutyl GSL (4MSOB), (**c**) 8-methylsulfinyloctyl GSL (8MSOO), (**d**) 4-methylthiobutyl GSL (4MTB), (**e**) 7-methylthioheptyl GSL (7MTH), (**f**) 8-methylthiooctyl GSL (8MTO), (**g**) indol-3-ylmethyl GSL (I3M). Rosette leaves (RL), cauline leaves (CL), primary stems (PS), lateral stems (LS), and siliques (Si). White and green bars represent the relative GSL content in WT and *sel1-10* plants, respectively. Data are shown as averages with error bars denoting SEM (n = 3). Asterisks indicate significant differences (Student’s *t*-test; * 0.05 < *p* < 0.1, ** *p* < 0.05) between WT and *sel1-10* plants. n.d., not detected. The chemical structures of the GSLs were obtained from KEGG Databases in DBGET (https://www.genome.jp/dbget/).

We harvested RL, CL, PS, LS, and Si separately and analyzed sulfate, cysteine, glutathione (GSH), and GSL in different parts of the *sel1-10* and WT plants (Figure 2, Figure 3 and Figure 4).

Sulfate content in the RL of *sel1-10* plants was 26% higher than that in the WT plants. Both WT and *sel1-10* plants accumulated a similar level of sulfate in CL, PS, and LS. In Si, the sulfate content of *sel1-10* plants was 61% of that in the WT plants. These results indicated that the distribution of sulfate was modulated in *sel1-10* plants.

To examine the effects of modulated sulfate distribution in *sel1-10*, cysteine and GSH contents in WT and *sel1-10* plants were analyzed (Figure 3). Cysteine content was not significantly different between WT and *sel1-10* plants in all examined parts. The GSH content in Si of *sel1-10* plants was 29% lower than that in WT plants, suggesting that the dysfunction of SULTR1;2 affects GSH accumulation in reproductive tissues as observed in the seedlings [28]. GSH content in other parts of *sel1-10* plants was similar to that in the WT plants.

The following seven major GSLs were analyzed in both plants (Figure 4). These included six mGSLs: 3-methylsulfinylpropyl GSL (3MSOP), 4-methylsulfinylbutyl GSL (4MSOB), 8-methylsulfinyloctyl GSL (8MSOO), 4-methylthiobutyl GSL (4MTB), 7-methylthioheptyl GSL (7MTH), and 8-methylthiooctyl GSL (8MTO), and one iGSL, indol-3-ylmethyl GSL (I3M).

GSL levels were generally lower in RL, CL, PS, and LS of *sel1-10* relative to the same parts of WT plants (Figure 4). However, in Si, GSL levels did not significantly vary between *sel1-10* and WT plants, and some GSL levels were even higher in *sel1-10* plants relative to the WT plants, that is, the levels of MSOX GSLs and I3M were similar between *sel1-10* and WT plants, but the levels of MTX GSLs were higher in *sel1-10* plants relative to the WT plants (Figure 4).

**Figure 5:**
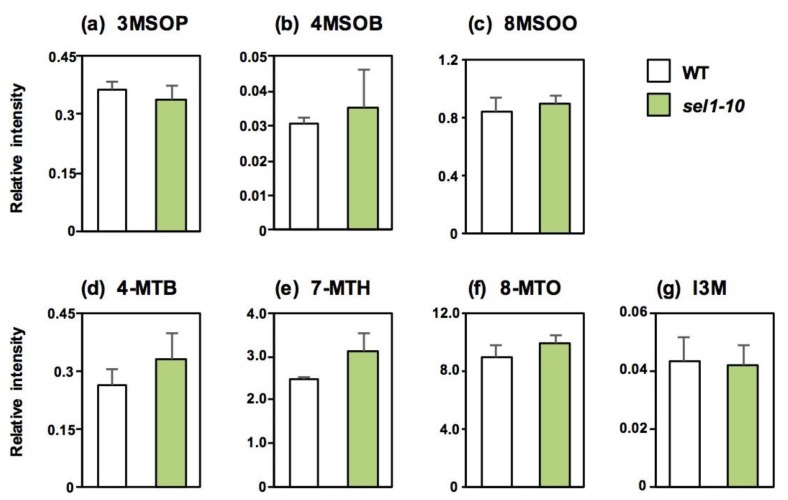
GSL accumulation in mature seeds of WT and *sel1-10* plants. GSL levels in seeds were determined by LC-MS. The relative amount was calculated as the ratio of peak height of each GSL to that of the internal standard (L(+)-10-camphor sulfonic acid) and then divided by the dry weight of the sample. (**a**) 3-methylsulfinylpropyl GSL (3MSOP), (**b**) 4-methylsulfinylbutyl GSL (4MSOB), (**c**) 8-methylsulfinyloctyl GSL (8MSOO), (**d**) 4-methylthiobutyl GSL (4MTB), (**e**) 7-methylthioheptyl GSL (7MTH), (**f**) 8-methylthiooctyl GSL (8MTO), (**g**) indol-3-ylmethyl GSL (I3M). White and green bars represent the relative GSL content in WT and *sel1-10* seeds, respectively. Data are shown as averages with error bars denoting SEM (n = 3). Statistical analysis was performed with Student’s *t*-test between WT and *sel1-10* plants, but any significant differences were not detected.

Because GSL levels in Si were not affected in *sel1-10* plants except for 4MTB and 7MTH, GSL levels in mature dried seeds were analyzed to determine the effects of reduced sulfate uptake (Figure 5). Seeds contained much higher levels of MTX GSLs and 8MSOO and lower levels of I3M compared to other vegetative tissues in both plant lines, which is consistent with previous studies [8,9]. In seeds, GSL levels did not significantly vary between *sel1-10* and WT plants.

## 3. Discussion

Growth phenotypes of mature *sel1-10* plants have not been well studied as regards their aerial part. The sulfate uptake rate in *sel1-10* plants was almost half of that in WT plants under both +S and –S conditions at the seedling stage [27,29]. In addition, the biomass and the levels of sulfate and GSH in *sel1-10* seedlings were significantly lowered relative to those in the WT plants under both +S and –S conditions [27,28,29]. Similar growth retardation in mature *sel1-10* plants observed in Figure 1 is assumed to be due to the reduction in sulfate uptake in *sel1-10* plants.

It is known that GSL accumulation is differentially regulated in plant parts and S status [8,9,13,21,22]. In our analysis, the levels of MSOX GSLs and I3M in the leaves and stems of *sel1-10* plants were significantly lower than those in the WT plants (Figure 4), in agreement with the –S-induced-like phenotypes observed in *sel1-10* seedlings [27,28]. In contrast, the GSL levels in Si and Se of *sel1-10* plants were similar or higher than those in WT plants (Figure 4 and Figure 5). Considering the previous theory that GSLs accumulated in the seeds provide an S source for seedling growth [8,9,10,21], GSL accumulation should be prioritized in reproductive tissues even when the S supply is limited in *sel1-10* plants. Plants should have adapted to fluctuations in S availability by using GSLs as S storage substances in reproductive tissues. GSLs can be considered as a beneficial S storage compounds because of the relatively high molecular weight, that enable them to reduce osmotic pressure in the seeds. Additionally, GSLs can be a source of carbon and nitrogen, especially in the case of long-chain mGSLs highly accumulated in the seeds, and can also act as the defense compounds to protect the seeds from diseases or predators [11,21].

Unexpectedly, the levels of MTX GSLs were higher in Si of *sel1-10* plants compared to those in the WT plants, while MSOX GSL and iGSL levels in Si were similar between *sel1-10* and WT plants (Figure 4). Taking into account that GSL levels in Si were the sum of the levels in silique tissues, including the developing seeds, and all samples were collected on the same date, the increase of MTX GSLs could be because of the acceleration of seed maturation in *sel1-10* plants. In general, nutrient stress accelerates bud appearance and subsequent development of the siliques and seeds in *Arabidopsis* [30,31]. Considering that MTX GSLs in the seeds are continuously increased during the seed maturation period [8,9], the timing of flowering and seed development may occur earlier in *sel1-10* than in the WT plants. Lower levels of sulfate and GSH in Si of *sel1-10* plants relative to those in the WT plants also support this assumption (Figure 2 and Figure 3).

Several maternal tissues have been suggested as source tissues for seed GSLs, including the leaves and siliques [10,11]. Although the GSL transport machinery in whole plants is not fully understood [10,11,12,32], GSL transporters, GTR1 and GTR2, that belong to the NRT/PTR family have been characterized for their roles [32,33]. In the double disruption lines of GTR1 and GTR2, most GSLs were not found in the seeds, whereas, mGSLs were highly accumulated in rosette leaves and siliques [32,33]. This suggested that seed GSLs are mostly transported from the source tissues. Decreased GSL levels in vegetative tissues and the maintenance of GSL levels in the seeds suggested that GSL transport to the seeds was not restricted or was even accelerated in *sel1-10* plants. 

In conclusion, we found that GSL levels of the MSOX group were decreased in the leaves and stems, whilst all GSL were found to be maintained in the seeds in *sel1-10* plants. This shows that accumulation of mGSL characterizes the reproductive tissues, thus indicating that mGSL are destinated to store in the seeds in order to support the initial growth of the next generation.

## 4. Materials and Methods

### 4.1. Plant Materials and Growth Conditions

*Arabidopsis thaliana* were cultured in a growth chamber controlled at 23 ± 2 °C under constant illumination (40 µmol m^−2^ s^−1^). The *sel1-10* mutant, carrying a T-DNA insertion in the ninth exon of SULTR1;2 (At1g78000) [28], and the background Wassilewskija (Ws-0) wild-type plants (WT) were used as plant materials. Seeds of WT and *sel1-10* were sown on vermiculite as growth substrate supplemented with MGRL mineral nutrient media in 5 × 5 × 5 cm plastic pots [34,35]. After germination, the number of plants was adjusted to three plants per pot. Plants were grown for 6 weeks and the different parts of the plants, rosette leaves (RL), cauline leaves (CL), primary stems (PS), lateral stems (LS), and siliques (Si) were harvested separately from each pot and weighed for the fresh weights. Mature dried seeds collected from the former generation were used for the analysis. Right after harvest, plant tissues were frozen in liquid nitrogen, freeze-dried, ground into a fine powder using a Tissue Lyser (Retsch, Germany), and used for each metabolite analysis. Three independent samples for each part were used for metabolite analysis. 

### 4.2. Measurement of Glucosinolates

Three milligrams of the plant powder was extracted with 300 µL of ice-cold 80% methanol containing 2 µM L(+)-10-camphor sulfonic acid (10CS, internal standard, Tokyo Kasei, Japan) using a Tissue Lyser. After homogenization, cell debris was separated by centrifugation (15,000 rpm, 10 min, 4 °C), and the supernatants were evaporated with a centrifugal evaporator (CVE-3110, EYELA, Japan) connected to a high vacuum pump (DAH-60, ULVAC, Japan) and a cold trap (UNI TRAP UT-1000, EYELA). Dried supernatants were dissolved into water, filtered with Millex-GV filter units (Millipore, USA), and analyzed by a high-performance liquid chromatograph connected to a triple quadrupole (LC-QqQ)-MS (LCMS8040, Shimadzu, Kyoto, Japan) using L-column 2 ODS (pore size 3 µm, length 2.1 × 150 mm, CERI, Japan). The mobile phase consisted of solvent A (0.1% formic acid, Wako Pure Chemicals, Osaka, Japan) and solvent B (0.1% formic acid in acetonitrile, Wako Pure Chemicals, Osaka, Japan). The gradient elution program was as follows with a flow rate of 0.3 mL/min, 0–0.1 min, 1% B; 0.1–15.5 min, 99.5% B; 15.5–17 min, 99.5% B; 17–17.1 min, 1% B; and 17.1–20 min, 1% B as described previously [36]. For the MS, electrospray ionization mass spectrometry technique in negative ionization mode was used. The ionization parameters were as follows: the nebulizer gas flow was 1.5 L/min, the CDL temperature was 250 °C, heat block temperature was 400 °C. All GSLs were detected with optimized selective reaction monitoring transitions in negative ionization mode as follows (precursor ion [*m/z*]/product ion [*m/z*] scores are shown): 3MSOP GSL: 422.02/358.02, 422.02/96.9, 422.02/95.9; 4MSOB GSL: 436.05/96.9, 436.05/96.0, 436.05/177.9; 8MSOO GSL: 492.1/428.1, 492.1/96.9, 492.1/95.9; 4MTB GSL: 420.04/96.9, 420.04/95.9, 420.04/74.9; 7MTH GSL: 462.09/96.9, 462.09/95.9, 462.09/74.9; 8MTO GSL: 476.11/96.9, 476.11/95.9, 476.11/74.9; I3M GSL: 447.05/96.9, 447.05/95.9, 447.05/74.9. MRM transitions were determined by using standard compounds (Cfm Oskar Tropitzsch GmbH, Marktredwitz, Germany) or a database (MassBank, http://www.massbank.jp). The relative quantities of GSLs were calculated as the ratio of peak height to the height of 10CS.

### 4.3. Measurement of Sulfate, Cysteine and Glutathione

One mg of the plant powder was extracted with 200 µL of 10 mM HCl. The cell debris was removed by centrifugation, and the supernatant was used for the analysis. The extracts were diluted 100 fold with extra pure water and analyzed by ion chromatography as described previously [29], using an eluent containing 1.9 mM NaHCO_3_ and 3.2 mM Na_2_CO_3_.

Cysteine and GSH contents were determined by monobromobimane (Invitrogen) labeling of thiol bases after reduction of the extracts with dithiothreitol (Nacalai Tesque) as described [13,28,29]. The labeled products were then separated by HPLC (JASCO, Tokyo, Japan) using the TSKgel ODS-120T column (150 × 4.6 mm, TOSOH) and detected with a fluorescence detector FP-920 (JASCO), monitoring for fluorescence of thiol-bimane adducts at 478 nm under excitation at 390 nm. GSH and Cys standards were purchased from Nacalai Tesque (Kyoto, Japan).

### 4.4. Statistical Analysis

The data were statistically analyzed using Student’s *t*-test with Microsoft Excel. Significant differences between WT and *sel1-10* in biological replicates are shown in each Figure.
